# Digitalization in the Emergency Department—An Interview Study of Nurses’ Experiences in Norway

**DOI:** 10.3390/nursrep14020106

**Published:** 2024-05-31

**Authors:** Ann-Chatrin Linqvist Leonardsen, Vivian Nystrøm, Renate Slang, Eilen Olsen, Anne Kristin Hole Trollnes

**Affiliations:** 1 Faculty of Health, Welfare and Organization, Østfold University College, 1757 Halden, Norway; vivian.nystrom@hiof.no (V.N.); renate.slang@hiof.no (R.S.); anne.k.trollnes@hiof.no (A.K.H.T.); 2 Department of Surgery, Østfold Hospital Trust, 1714 Grålum, Norway; eilen.olsen@so-hf.no

**Keywords:** emergency nursing, digitalization, qualitative interviews

## Abstract

Emergency departments (EDs) are overcrowded and linked to an increased risk of mortality and morbidity. Digitalization in EDs has been shown to increase effectiveness, reduce wait times, and improve performance and patient experience. The purpose of this study was to explore ED nurses’ experiences with digitalization in the ED. Interviews were conducted with eight ED nurses in a Norwegian hospital. Data were analyzed using Braun and Clarke’s six-step thematic analysis. Through analysis, three themes were identified, namely (1) consequences for patient safety, (2) influencing communication in the ED, and (3) impacting acute nursing. ED nurses experienced that the digital tools had increased patient safety through accurate documentation and providing a quick overview of the patient. However, digital tools were also seen as a threat to patient safety due to taking focus away from the patient. Digital tools were experienced to have negatively changed the communication both between personnel and between personnel and patients. Also, digital tools impacted the ED nurses’ professional role to a more digitalization-focused approach rather than a patient-oriented approach. These aspects must be included when planning the implementation of new digital tools in EDs in the future.

## 1. Introduction

Internationally, it has been claimed that appropriate technology provides solutions to improve healthcare services at an affordable cost [1]. Studies indicate that digitalization may lead to the standardization of patient care, prevention of medical errors, a better diagnosis process, higher patient safety, the digital readiness of healthcare workers, and the quality of healthcare in general [2]. The emergency department (ED) is one of the most crowded hospital units, where many patients with various medical conditions, including high-risk patients, are admitted [3]. Here, overcrowding is an acknowledged challenge, due to the gap between the need for emergency care and the hospital’s availability to provide the services needed [4]. Overcrowding may increase mortality and morbidity and lead to a decrease in the ability to provide emergency services in a timely manner [3]. In addition, the work of emergency clinicians is characterized by interruption and multitasking [5].

### 1.1. Literature Review

A 2022 literature review, including 25 articles, explored the available digital health technologies and their effectiveness in improving performance within this complex ED context [6]. The results indicated that digital tools and artificial intelligence were useful to detect, collect, and process data from patients. Digital tools were used to curb patients’ pain and anxiety and were assumed as effective tools for the clinical guidance of patients after discharge. Also, wearable tracking technology has shown promise for data-driven innovations in ED-clinician workflow [7]. A systematic scoping review of studies on handheld computer devices to support clinical decision-making in acute nursing indicated that these can be used effectively [8]. However, the authors underlined the need for more research.

Studies show that healthcare personnel’s knowledge impacts whether digital technology is adopted or not [9,10]. Also, their attitudes are important aspects in the acceptance and implementation of new technologies [11,12]. A study of urgent care clinicians’ perceptions of digital access to patients’ past medical history in the ED found that clinicians had a high level of technology acceptance, even if they also had several areas that needed improvement [13]. Farrell et al. [14] found that ED nurses reported iPhones as accessible and improving communication in the workplace. However, they were perceived as unprofessional when using the device together with patients and relatives. Lavin et al. [15] explored ED nurses’ experiences with using an electronic health record (EHR). Even if nurses found the EHR useful in enhancing patient safety, evaluating care quality, maximizing efficiency, and measuring staffing needs, they also indicated dissatisfaction with its design and cumbersome electronic processes.

### 1.2. Originality

Previous studies have provided an overview of available technologies in the ED [6], and explored the effects of wearable tracking devices [7] or handheld computer devices [8]. Studies exploring ED nurses’ perspectives are limited to focusing on iPhones [14] and EHRs [15]. However, studies focusing more broadly on digitalization in the ED from ED nurses’ perspectives are lacking.

### 1.3. Purpose

The purpose of this study was to explore ED nurses’ experiences with digitalization in the ED. This knowledge is essential when implementing new digital technologies in EDs, specifically for managers planning for such implementation.

## 2. Materials and Methods

### 2.1. Design

Qualitative studies are appropriate for the in-depth exploration of people’s experiences and perspectives [16]. As such, a qualitative design was chosen, using individual interviews with ED nurses in the period November 2022–January 2023.

The authors (all female) included an operating room (OR) nurse (MSc), a nurse anesthetist (Ph.D., professor, experienced with qualitative studies), an ED nurse (MSc), and two intensive care nurses (MSc, one with a PhD, experienced with qualitative studies). The study adheres to the Consolidated Criteria for Reporting Qualitative Research (COREQ) [17].

### 2.2. Setting and Participants

In Norway, EDs are most commonly manned with ED nurses: registered nurses (RNs) with a bachelor’s degree of 180 ECTS (European Credit Transfer and Accumulation System), intensive care or acute nurses with an additional education of 90 ECTS, and physicians. The study was conducted in a medium-sized hospital with a catchment area of 320,000 inhabitants. The hospital is one of the most modern hospitals in Europe and is on the leading edge of service-oriented innovation, including digital technology. A purposive sampling strategy [16] was used to identify participants who could provide in-depth information about digitalization in the ED. Managers from the ED were asked to recruit ED nurses with variations in gender, age, and years of clinical experience. The inclusion criteria were a minimum of 50 percent clinical work and having worked in the ED during the past year. After having agreed to participate, the last author contacted the ED nurses to agree on a time and place for an interview.

### 2.3. Data Collection

An interview guide was developed based on previous research on digitalization in the ED [6] and several discussions between all the authors (see Box 1 below).

Box 1 **Interview guide.**

Which equipment or tools do you associate with “digital” in your work?

- Have these tools changed your work pattern or how you work?
- What are the advantages of such digital tools?
- What are the disadvantages of such digital tools?

Have you received sufficient training and/or education to be able to use the tools?

Do you have any thoughts on how the use of digital tools may impact communication with patients?

Do you have any thoughts on how the use of digital tools may impact communication with relatives?

Do you have any thoughts on how the use of digital tools could have made your work more effective?

Is there anything else you would like to add regarding the use of digital tools in your work?

The guide was piloted by two nurses working in the ED, one intensive care nurse and one RN, both with several years of experience in the ED. The questions were deemed relevant and comprehensive; hence, the interview guide was not revised.

The first author conducted all the interviews in a meeting room at the hospital, in an office at the university, or digitally on a secure platform (Teams^®^, version 23247.720.2421.8365), with both sound and video. The audio of the interviews was digitally recorded and transcribed verbatim by an external transcriber who had signed a non-disclosure agreement. The aim was to include ten ED nurses. However, after eight interviews, the researchers saw similar instances over and over again and became empirically confident that a category was saturated [18].

### 2.4. Analysis

An inductive and thematic analysis inspired by the six-step approach of Braun and Clarke [19] was used to analyze the data. In step 1, all authors read all transcripts to familiarize themselves with the data. Then, in step 2, the last author individually coded the transcripts line by line, identifying key elements that could provide information about the study’s purpose. These codes were then reviewed by the first author to sense-check ideas and explore multiple assumptions or interpretations of the data [20]. In step 3, all authors discussed these codes and their interpretations of patterns of meaning across the dataset, representing preliminary themes. In step 4, the first and last author revisited the complete dataset, comparing it with the preliminary codes and also discussing whether their personal assumptions or experiences may have impacted their interpretations. This resulted in a total of seven themes, namely “Patient safety”, “Communication”, “Documentation”, “Perspectives on digital tools”, “Patient and relatives”, “Training”, and “Utilization of digital tools”. In step 5, these themes were reviewed in group discussions between all authors, and the final themes were defined and named. The authors’ own backgrounds, experiences, and assumptions were included in the iterative process from codes to themes, to increase the trustworthiness of the interpretations. Step 6 consisted of writing up the results based on the themes identified.

### 2.5. Ethics

All participants provided willing, informed, written consent to participate. The study was conducted in line with the ethical guidelines for research in the Declaration of Helsinki [21]. The study was approved by the Norwegian Agency for Shared Services in Education and Research (SIKT, project no. 538537). According to Norwegian legislation, no ethical approval was needed.

## 3. Results

### 3.1. Participant Demographics

In total, seven acute nurses and one intensive care nurse with many years of experience in the ED were interviewed. The participants’ ages ranged from 27 to 61 years (median 37.5 years), and their experience as an ED nurse ranged from 6 months to 22 years (median 2.8 years). The participants’ background information is not combined due to confidentiality issues. Representative quotes are marked as ED numbers 1–8, as appropriate.

The participants included several different tools in their description of “digital tools” in the emergency department: The electronic journal record Metavision^®^; a digital communication and logistics system (Imatis^®^); the medical journal record (DIPS^®^); a system for image diagnostics (PACS^®^; version 5.26.0-1173); a system for reviewing Electrocardiograms (ECGs) (Compax^®^); and mobile phones with various applications (apps), including DNV Imatis^®^, app for blood collection, blood transfusion apps, and several others were included. Through analysis, three themes were identified, namely (1) consequences for patient safety, (2) influencing communication in the ED, and (3) impacting acute nursing.

### 3.2. Consequences for Patient Safety

On one hand, the ED nurses experienced that the digital tools led to increased patient safety. Most of the participants stated that digital tools somehow had made their work “easier” on a daily basis. This was related to digital tools being easily accessible. Vital parameters were automatically gathered, and the apps, e.g., helped them administer blood products more safely without having other nurses control the administration This again reduced pressure on the nurses who then could focus more on the patients. Moreover, digital documentation was assumed to be more understandable than the former handwriting, which again ensured correct documentation and interpretation. Also, several of the ED nurses referred to “the system” ensuring that one could not proceed if anything was missing and, therefore, that all vital information was included. ED 7 stated:

“The paper journals were very chaotic…Things took much longer time…Now, all have the same access to information all the time, and one can work on the same patient in the same curve at the same time. It’s more effective”.

To the ED nurses, these aspects implied increased patient safety. This was also illustrated by ED 8, who said:

“With the blood-sample app, I can just scan the patient’s bracelet, and I don’t have to ask for personal identification again. We can take blood-samples while the patient talks to the doctor, and don’t have to interrupt. And that safety, that you actually have the right patient…”

In addition, many of the ED nurses underlined that the digital tools enabled them to get a quick overview of the patient. Also, since many of the tools were linked to each other, the ED nurses got alarms on their mobile cellphones if vital parameters were deteriorating, e.g., cardiac arrest.

On the other hand, the ED nurses also experienced that the digital tools impaired patient safety. This was due to “disturbing alarms” and “focus on the screen” stealing focus away from the patient, and some digital tools being “time-consuming”. Also, e.g., ED 2 had experienced getting measurements on the wrong patient, which could have led to wrong interventions.

### 3.3. Influencing Communication in the ED

All the ED nurses stated that digital tools had changed the communication between personnel and between personnel and patients. They reported that most of the communications between personnel were through digital messages. To the ED nurses, this had negatively impacted professional communication. Where they previously had interprofessional discussions, they now mostly communicate digitally. To ED 6, this made especially new nurses vulnerable for not being “internally educated”, due to not being informed by, e.g., the doctors, and just “executing orders”. ED 3 also underlined that this did not ensure that the message was received. ED 1 described the change like this:

“The communication has become worse, and in some cases catastrophically worse…Because the doctor sits in another room, writing messages…and the nurse is assumed to catch this, who has far too much to do, and of course is not accessing the data to receive messages from the doctor in the next room…”

This was also underlined by several of the other ED nurses. Also, digital communication was used in handovers to other wards. This was assumed to be a risk that not all vital information was transferred and received due to personnel not “sitting at the computer all the time, waiting for messages” (ED 5). In addition, digital reports did not allow for follow-up questions or clarifications.

Further, the ED nurses experienced that digital tools had changed communication with patients and relatives. On one hand, digital tools had provided ED nurses with access to more information, enabling them to communicate more with the patient and relatives. Also, the ED nurses experienced that the digital tools made them more accessible to relatives, mainly due to always having a mobile cellphone in their pocket. On the other hand, digital tools were experienced to limit communication with patients because the ED nurses were “always occupied” with the tools. ED 8 stated:

“…the patient is always connected to the monitor, and there’s less supervison…We are more seldom bedside, to ask how the patient is. We rather look at the screen…”

### 3.4. Impacting Acute Nursing

Most of the ED nurses underlined that digitalization in the ED impacted their professional role and performance in acute nursing. First, digital tools were reported to change the ED nurses’ nursing approach to be somewhat less patient oriented. For example, ED 4 claimed:

“One uses more time to keep updated on all the different softwares, right, than is actually used on patient-related treatment that benefit our patient.”

This impression was supported by all of the ED nurses, who underlined that the digital tools required time both to learn initially and to use in their daily work. This time was related to onlogging issues, error warnings that needed to be solved, and various systems leading to double documentation. In an overcrowded ED with multiple parallel tasks, this was assumed to “instrumentize” the patient. Also, according to the ED nurses, digitalization had led to a situation where patients were always monitored, digitally, which allowed for ED nurses to be more absent.

Secondly, digitalization was assumed to make the ED nursing role more “independent” or “lonely”, changing the dynamics in the collaboration with other healthcare professionals. For example, ED 3 described:

“…one work so much on the computer, versus working clinically with patients…and one remain on separate offices, linked to that one specific location than before.”

Third, ED nurses also reported that this change had led to confusion about roles in an emergency team, and unclarities about which tasks belonged to which professional. ED 8 elaborated:

“The collaboration suffers from not seeing each other so often anymore…The communication is impaired, leading to more distance between the different professionals”

## 4. Discussion

### 4.1. Discussion of Main Findings

This study provides insight into ED nurses’ experiences with a broad sample of digital tools implemented in the ED. The results show that digitalization in the ED was assumed to both increase and decrease patient safety, that digital tools changed the way professionals communicated both interprofessionally and with patients and relatives, and that ED nurses perceived that digitalization impacted their professional role in acute nursing.

On one side, the ED nurses in this study experienced that the digital tools led to correct documentation, accessible information, and a more effective work process. To them, this represented increased patient safety. This positive effect of digitalization in the ED has been underlined in several previous studies [6,7,8,15]. However, few of these studies were based on the perspectives of ED nurses and beyond specific digital tools, such as wearable sensors or handheld computers. Also, the negative impact of digital tools on patient safety, as reported in this study has been indicated in previous studies, e.g., in an operating room context [22,23]. Here, digital tools were reported to “steel focus” from the patient, and, therefore, decreased patient safety, as also reported by the ED nurses. However, e.g., Tse et al. [24] found no effect of digitalization on personnel’s vigilance toward the patient. These contradictions may be caused by the lack of information about patient outcomes due to or despite digitalization. As such, Klausen et al. [25] suggest that data-driven tools providing systematic feedback on patients’ outcomes may be valuable for improving emergency service quality and patient safety.

On the other side, the ED nurses in this study underlined the negative impact of digitalization on communication, both between personnel and between personnel and patients or relatives. Previous studies partly contrast this, indicating that electronic health records lead to better communication between healthcare personnel, improved accessibility, and support for quality of care [26]. However, some studies also support our findings, underlining, e.g., a decreased frequency of direct communication among healthcare professionals [27] or misconceptions [28]. Also, Vos et al. [29] found that the reduced necessity for face-to-face communication saved time but was experienced as hindering collective responsibility for a smooth workflow. Patients in EDs are already vulnerable, being acutely admitted to the hospital [30]. Blackburn et al. [31] found that effective communication resulted in ED patients feeling informed and knowledgeable about treatment and care. However, this relied on regular interactions with healthcare personnel. In contrast, poor interpersonal communication has been associated with frustration and dissatisfaction in ED patients [32].

Finally, the ED nurses experienced that digital tools had changed acute nursing. A systematic review of studies focusing on the impact of health information technology on nurses’ time [33] found that the ‘systems’ increased nursing documentation time. However, many ‘systems’ also resulted in nurses spending more time in direct care and “value-adding” activities. The negative experience reported in our study may be impacted by the context, including the overcrowded and busy ED, where ED nurses are continuously interrupted and multitasking, maybe resulting in a feeling of not being able to provide appropriate and timely services [3,4,5]. This may, again, impact their feeling of professionalism. A systematic review of studies on barriers and facilitators to utilizing digital health technologies by healthcare professionals [34] found that infrastructure and technical issues, psychological barriers, and workload-related concerns were barriers to adopting digital health technologies. Facilitators reported perception of usefulness and willingness to use, as well as various stakeholders’ incentives.

The ED nurses in our study experienced both positive and negative effects of digitalization in the ED. This division into positive and negative effects has also been underlined in other studies [33,35]. According to the World Health Organization [36] digital innovation is happening at an unprecedented scale—not only in Eds—hereby harnessing the power of such technologies and health innovation to accelerate global attainment of health and wellbeing [36]. However, it seems like there is a need to increase ED nurses’ knowledge about the effectiveness of such tools to achieve more positive attitudes and, therefore, acceptance [9,10,11,12]. Digital literacy has been defined as “the ability to access, manage, understand, integrate, communicate, evaluate and create information safely and appropriately through digital technologies for employment, decent jobs and entrepreneurship” [37]. Such competence is stated to play an essential role for health-science students to understand the ethical implications of using technology in healthcare and its potential risks [38]. A 2020 study on digital literacy levels among healthcare personnel found that younger persons (<50 years) were more frequent users of computers, mobile devices, email, the Internet, and social media when compared with older persons (>50 years) [39]. Aydinlar et al. [40] underlined the importance of defining the level of computer skills among students starting health-based education and shaping the curriculum by determining which domains are weak, and also discussing various digital literacy topics.

### 4.2. Methodological Considerations

This study has some limitations. First, the study was conducted in one Norwegian hospital only, and the findings may, therefore, not be transferable to other settings. Moreover, the sample was relatively small, including only eight ED nurses. However, we assumed the information power of the data to be rich [16].

Second, the participants were familiar to the interviewer, as former colleagues in the ED. This may represent a researcher bias. However, the issues presented are not sensitive or confidential, and the interviewer perceived that the ED nurses openly shared their experiences, perspectives, and reflections.

To increase the trustworthiness of the study, all authors with various professional backgrounds were involved at different stages of the analysis process. Presenting the results to the participants could have increased the dependability of our interpretations. However, there is no scientific consensus on whether this is appropriate.

## 5. Conclusions

This study adds to the limited existing knowledge about ED nurses’ perspectives on digitalization in the ED. Our results indicate that digitalization impacts patient safety, communication, and acute nursing in the ED. ED nurses’ knowledge and attitudes are essential for the successful implementation of digital tools in the ED. Their experiences underline the importance of focusing on the development of digital literacy, both during education and as part of life-long learning.

Further studies should explore the impacts of digitalization on various professionals’ perspectives, as well as on patient outcomes in an ED setting.

### Clinical Implications

Based on our results, we suggest that ED managers conduct a risk analysis when implementing new digital technology, regarding whether this can impact the focus on the patient and communication between all stakeholders. Also, proper education and training must be ensured for all professionals involved in using the technology. The EDs should critically assess alarm limits and the consequences of adjusting these. Also, the issues stated in this study should be discussed in interprofessional meetings. The issues are also relevant to policymakers, underlining the effects on human interaction from digitalization.

## Data Availability

The data are available upon reasonable request to the first author.

## Public Involvement Statement

There was no public involvement in any aspect of this research.

## Guidelines and Standards Statement

The study adheres to the Consolidated Criteria for Reporting Qualitative Research (COREQ).
